# Unraveling the role of Calcium ions in the mechanical properties of individual collagen fibrils

**DOI:** 10.1038/srep46042

**Published:** 2017-04-05

**Authors:** Xiangchao Pang, Lijun Lin, Bin Tang

**Affiliations:** 1 Department of Biomedical Engineering, Southern University of Science and Technology, Shenzhen 518055, P. R. China; 2 Department of Physics and Key Laboratory of Artificial Micro- and Nano-structures of Ministry of Education, School of Physics and Technology, Wuhan University, Wuhan 430072, P. R. China; 3 Department of Orthopedics, Zhujiang Hospital, Southern Medical University, Guangzhou 510515, P. R. China; 4 ShenZhen Key Lab of Nanoimprint, Shenzhen, P. R. China

## Abstract

Collagen, the dominating material in the extracellular matrix, provides the strength, elasticity and mechanical stability to the organisms. The mechanical property of collagen is mainly dominated by its surrounding environments. However, the variation and origin of the mechanics of collagen fibril under different concentrations of calcium ions (χ_Ca_) remains unknown. By using the atomic force microscopy based nanoindentation, the mechanics and structure of individual type II collagen fibril were first investigated under different χ_Ca_ in this study. The results demonstrate that both of the mechanical and structural properties of the collagen fibril show a prominent dependence on χ_Ca_. The mechanism of χ_Ca_-dependence of the collagen fibril was attributed to the chelation between collagen molecules and the calcium ions. Given the role of calcium in the pathology of osteoarthritis, the current study may cast new light on the understanding of osteoarthritis and other soft tissue hardening related diseases in the future.

Calcium is one of the most important ions in human body and plays a critical role in certain physiological functions. It has been proven that several human diseases are related with the disturbance of calcium, e.g., osteoarthritis, cardiac arrest, respiratory arrest, cataract and asteroid hyalosis^1^. According to the compositional analysis, Jubeck *et al*. found that the abnormal calcium enrichment was in up to 60% osteoarthritic articular cartilage (AC)^2^. In order to reveal the relationship between the calcium and osteoarthritic AC, researchers have investigated the effect of calcium on the proteoglycans, collagen and chondrocytes. These studies indicated that calcium plays a pivotal role in the death of chondrocytes, proteoglycans can be treated as a calcium-concentrating agent, and collagen also can bind calcium ions^3^,^4^. Although the role of calcium in the AC is extensively studied, there is a scarcity of studies about the influence of calcium on the mechanics of individual collagen fibril, which is the main composition of AC.

Collagen is one of the most abundant proteins in human body, which holds the whole body together^5^,^6^. It extensively exists in the bones, muscles, skin, blood vessels and connective tissues, and provides the strength, elasticity and mechanical stability to the body. For its key role in the body, collagen has attracted huge interests from both theoretical and practical points of view. Collagen fibril, the main form of collagen, consists of collagen molecules. Collagen molecules has a molecular structure that is composed of triple helix of left-handed polypeptide chain known as tropocollagen. In other words, collagen fibril can be treated as the linear aggregates of tropocollagen. The typical diameter and length of collagen molecule are ~1.5 nm and ~300 nm, respectively^7^. They generally self-assemble into a cross-striated collagen fibril with a D-band periodicity of about 67 nm. Such a so-called “quarter-staggered” structure creates the periodic gap (about 0.54 D) and overlap (about 0.46 D) regions where the gap regions have 20% less packing density than that of the overlap regions^8^,^9^. More than 20 genetically distinct collagens are found in animal tissue. Type I, II, III, V and XI collagens can form fibrils, and have the D-band periodicity^10^. Type I collagen, consisted of two α1(I) chains and one α2(I) chain, is found throughout the body except in cartilaginous tissues. Type II collagen, consisted of three identical α1(II) chains, is found in the cartilage, developing cornea and vitreous humour. There are some differences in the amino acid composition of α1 and α2, but they are minor^11^. The mechanics of collagen fibril as well as collagen molecules have been also extensively investigated^7^,^12^,^13^,^14^,^15^. However, how external ions (i.e., calcium ions) affect both structural and mechanical properties of individual collagen fibril is still ambiguous, especially under the nanoscale level.

Atomic force microscopy (AFM) based nanoindentation has been well established as a powerful technique for the investigation of the mechanics at the nanoscale level^9^,^16^,^17^,^18^,^19^. To have a better understanding of the effect of abnormal calcium homeostasis on soft tissues’ mechanics, we attempted to employ AFM-based nanoindentation to study the mechanical behavior of type II collagen fibril extracted from AC. The structural properties (D-band periodicity and the height difference between the gap and overlap regions) of type II collagen fibril were also investigated. It was found that the structural and mechanical behaviors of individual type II collagen fibril are significantly depending on the concentration of calcium ions (χ_Ca_). When 2 mM < χ_Ca_ <= 5 mM, the elastic modulus of individual collagen fibril increases with the increasing χ_Ca_; the structural characteristic of the collagen fibril, D-band periodicity, decreases with the increasing χ_Ca_; and another structural characteristic, the height difference between overlap and gap regions, increases with the increasing χ_Ca_. With further increase of χ_Ca_ (>5 mM), the elastic modulus and D-band periodicity of the collagen fibril level off; while the height difference between overlap and gap regions remains increasing. The mechanism of χ_Ca_-dependence of the collagen fibril was attributed to the chelation between collagen molecules and the calcium ions. Given the relationship between the disturbance of calcium and the tissue stiffening, the presented study strongly suggests that the tissue stiffening found in various diseases should be due to the interaction between calcium ions and collagen fibril. To the best of our knowledge, the presented work should be the first systematic study on the role of calcium ions on mechanical and structural properties of individual type II collagen fibril. This study will provide fundamental understanding of the influence of calcium ions on the collagen fibril as well as the pathology of related diseases, and so possibly lead to new diagnosis techniques and clinical treatments.

## Results

### Mechanics of individual collagen fibril under different χ_Ca_

**Figure 1:**
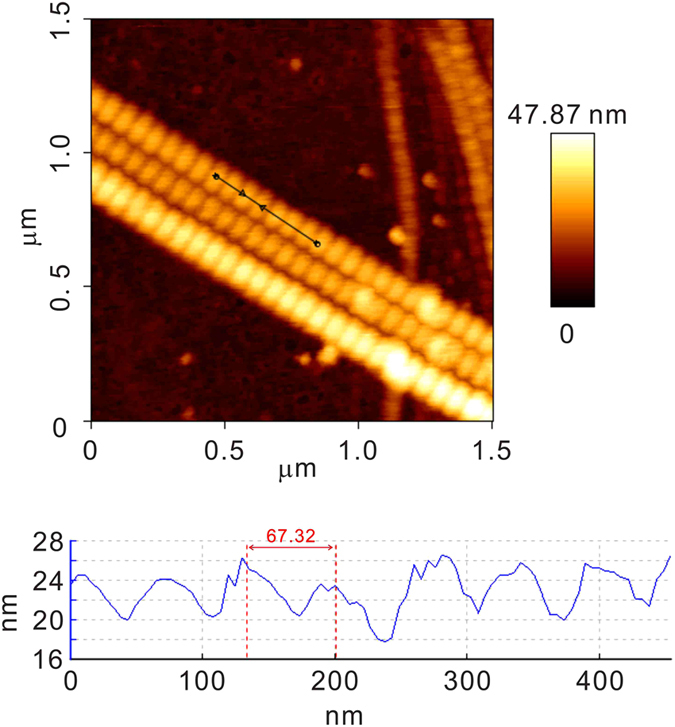
A typically topographical image (tapping mode in air) of type II collagen fibril deposited on a glass slide measured under the dehydrated state at room temperature (25 °C, RH = 20 ± 2%), with height profile along the dark line showing in the AFM image.

It is reported that five kinds of collagen (II, VI, IX, X and XI) have been identified in AC^20^. Type II collagen, a homotrimer composed of α1(II) chains, is the most abundant, which makes up about 90% of the total collagen in AC^21^. Therefore, collagen mainly investigated in this study is the type II collagen. In order to locate the collagen fibril, it is necessary to image the sample in advance of the nanoindentation measurement. Moreover, AFM images can provide a cross-check of the collagen fibril investigated in this study. Figure 1 shows a typical image of the collagen fibril obtained under the tapping model at room temperature (RT, 25 °C). The morphology of the type II collagen fibril shown in Fig. 1 is consistent with previous studies, especially the regular D-band periodicity, which is the typical characteristics of collagen fibril. The length of the D-band periodicity is also in agreement with previous studies on the type I collagen fibril^17^,^20^.

**Figure 2:**
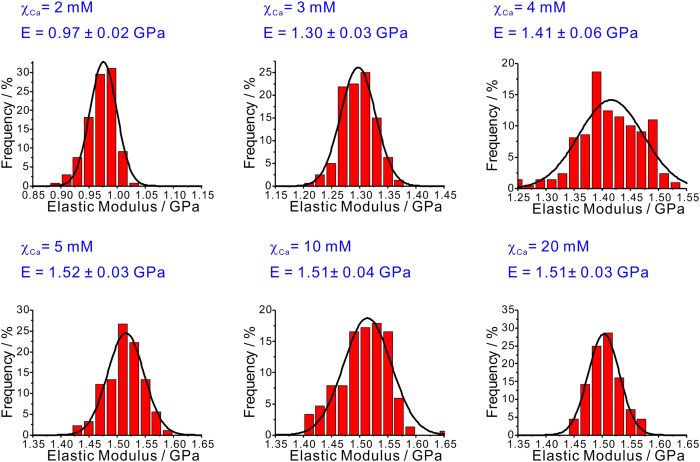
The frequency histograms with the same bin of 0.02 GPa of elastic modulus for the individual type II collagen fibril at different concentrations of calcium ions (χ_Ca_). The histograms were also fitted by the Gaussian curves (black curve). The mean values with the standard deviation of the elastic modulus of the individual type II collage fibrils at different χ_Ca_ were also shown.

Having successfully obtained the topology of the individual type II collagen fibrils, it was then possible to investigate their mechanical properties. The frequency histograms of elastic modulus of the individual type II collagen fibril under different χ_Ca_ were shown in Fig. 2. The data were collected from the nanoindentation measurements on more than 95 individual collagen fibrils for each group. The histograms of the measured elastic modulus were fitted by the Gaussian curves.

As illustrated in Fig. 2, the elastic modulus of the individual type II collagen fibril shows a remarkable dependence on χ_Ca_. When χ_Ca_ gradually increases from 2 mM to 5 mM, the elastic modulus of the individual collagen fibril shows a salient increase from 0.97 ± 0.02 GPa to 1.51 ± 0.03 GPa. However, when χ_Ca_ >5 mM, no evident variation of the elastic modulus for the individual collagen fibril is observed (see Fig. 2). The present data are also in the same order of magnitude with previous studies on the type I and type II collagen fibril (0.6 GPa to 7 GPa)^22^.

**Figure 3:**
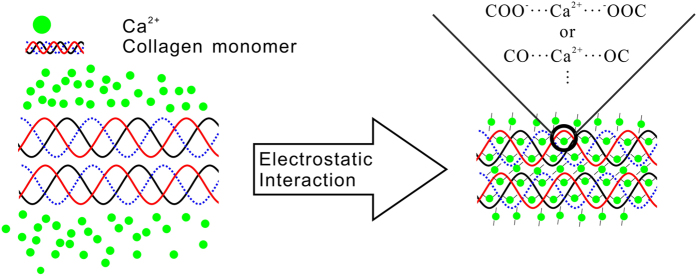
The schematic diagram of the interaction between calcium ions and collagen molecules. For clarity, the three identical α1(II)-chains of collagen molecules are drawn in different color lines. The –COO^−^ derived from the ionization of the carboxyl groups of Glu and Asp in α1(II) chains.

To elucidate the difference in the elastic modulus of individual type II collagen fibril under different χ_Ca_, it is better to check its chemical structure. Just as stated above, type II collagen fibril is composed of three identical α1(II)-chains. In other words, there are many charged and polar groups among the collagen fibril. The carboxyl groups (-COOH) of the Glu and Asp make up about 9.5% of the amino acid residues of α1-chain. In the neutral solution, more than 99% of these carboxyl groups ionized to -COO^−^, which favors chelation of calcium ions to form -COO^−^…Ca electrostatic bonds^23^,^24^. Therefore, it can be expected that the calcium ions prefer to bind these negatively charged groups in the collagen fibril due to the electrostatic interaction (see Fig. 3). This assumption is supported by the study of Rhee *et al*.^24^, who proposed that the red shifts of the carboxylate band in the type I collagen fibril originated from the chelation with calcium ions (Supplemental Fig. 1). Besides the -COO^−^, Cui *et al*. and Kawska *et al*. found that carbonyl oxygen in the type I collagen fibril can also chelate with calcium ions by forming the Ca…O electrostatic bonds^23^,^25^,^26^.

By chelating with the negatively charged groups (carbonyl and carboxyl groups) in the collagen molecules to form chelate rings, calcium ions can tightly couple with the collagen fibril. On the other hand, the chelation happened among the collagen molecules increases the degree of the fibril crosslinking (see Fig. 3). The enhanced fibril crosslinking will then lead to a compact structure, which was observed in the structural analysis results shown in later section. It is consistent with the finding of Cui *et al*. that the initially loose cluster structure of the type I collagen molecules formed into a more compact structure induced by the enhanced crosslinking^23^,^25^.

According to the Petruska–Hodge model^27^, the overlap region would be stiffer than the gap regions due to the compact packing of collagen molecules. Yu *et al*. and Grant *et al*. found that the overlap region of the type I collagen fibril is elastically higher (almost ∼100% stiffer) than that of the gap region by using the nanoindentation method^8^. Therefore, in the range of 2–5 mM, the increasing elastic modulus of the individual type II collagen fibril can be attributed to the enhanced crosslinking among the collagen molecules induced by calcium ions. As further increase of χ_Ca_, it is believed that the chelation between calcium ions and collagen molecules is nearly saturated. In other words, the crosslinking degree of type II collagen fibril is not affected by the further addition of calcium ions. Therefore, the elastic modulus of the individual collagen fibril does not vary with the further increase of χ_Ca_, and levels off at 1.51 GPa (χ_Ca_ > 5 mM).

The body of a healthy adult contains about 25,000 mmol of calcium, of which >99% is part of bone mineral and <1% is in the extracellular fluid^28^. The normal serum calcium concentration is maintained within the narrow range of 2–2.75 mM. However, the elastic modulus of the individual collagen fibril increases from 0.97 ± 0.02 GPa at χ_Ca_ = 2 mM to 1.30 ± 0.03 GPa at χ_Ca_ = 3 mM. This indicates that a slightly abnormal variation of calcium concentration can lead to an obvious increment (~34%) on the nanostiffness of the collagen. It is also confirmed that maintaining the serum calcium within its normal range is rather important^28^.

### Morphological variation of individual collagen fibril under different χ_Ca_

**Figure 4:**
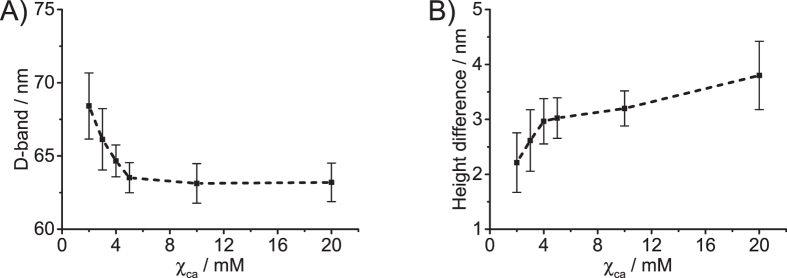
Variations of D-band periodicity (**A**) and the height difference between overlap and gap regions (**B**) for the individual type II collagen fibril at different concentrations of calcium ions (*T* = 25 °C, RH = 20 ± 2%). The mean value and standard deviation are represented by the filled square and short line, respectively. The dotted line is only a guide to the eyes. The frequency histograms of the D-band periodicity and the height difference of collagen fibrils at different χ_Ca_ are also shown in Supplemental Figure 2 and Supplemental Fig. 3, respectively. The D-band periodicity and the height difference was obtained by the analysis of the AFM image of the individual collagen fibril under different χ_Ca_, e.g., Fig. 1. The collagen fibrils used for the analytical analysis must meet the following criteria: (1) the collagen fibrils display regular D-band periodicity; (2) there are no overlaps with other collagen fibrils.

The naturally occurring crosslinking among the collagen molecules plays a crucial role in the formation of the stable collagen fibril. With the introduction of calcium ions, the crosslinking among the collagen molecules is further enhanced. In other words, the balance of the original crosslinking among the collagen molecules is disturbed by the introduction of calcium ions. Therefore, it can be expected the structural characteristics of the collagen fibril, such as D-band periodicity and height difference between overlap and gap regions, should be affected by the presence of calcium ions. In order to prove this assumption and further verify the influence of calcium ions on the mechanics of the individual collagen fibril, the images of the individual type II collagen fibril were analyzed. Figure 4 shows the variation of D-band periodicity (A) and height difference between overlap and gap regions (B) for individual type II collagen fibril at different χ_Ca_.

As shown in Fig. 4A, when χ_Ca_ gradually increases from 2 mM to 5 mM, the D-band periodicity of the type II collagen fibril shrinks from 68.4 ± 2.3 nm to 63.5 ± 1.0 nm. With further increase of χ_Ca_ (>5 mM), the D-band periodicity is no longer dependent on χ_Ca_, and it levels at 63 nm. However, the height difference between overlap and gap regions gradually increases from 2.2 ± 0.5 nm to 3.8 ± 0.6 nm with the increase of χ_Ca_ from 2 mM to 20 mM (Fig. 4B).

There are many functional groups (-COO^−^, derived from the ionization of the carboxyl groups of Glu and Asp in α1(II) chains, and the carbonyl groups) which can chelate with the calcium ions in the type II collagen molecules. Thus, the collagen molecules can form interaction with calcium ions in various combinations (see Fig. 3). It is possible that one calcium ion can interact with two adjacent groups in one α1(II)-chain and creates an “ionic bridge”. To match with the length requirement of the “ionic bridge”, the α1(II)-chain in the collagen fibril could be shortened in some extent. As a result, the D-band periodicity would be affected by the shorted α1(II)-chains. Consequently, the gradual shrink of the D-band periodicity of the type II collagen fibrils with the increasing χ_Ca_ can be attributed to the chelation occurred between calcium ions and collagen molecules. As further increase of χ_Ca_ (>5 mM), there is almost no group to bind the calcium ions in the α1(II)-chains. In other words, the chelation is nearly saturated in the collagen fibrils. In this case, the D-band periodicity of the type II collagen fibril did not vary with the further increase of χ_Ca_ (>5 mM).

It is known that the packing density of collagen molecules is different in the overlap and gap regions^8^. The packing density of overlap region is 20% higher than that of the gap region. As a result of the different packing densities, the calcium induced crosslinking among the collagen molecules in the overlap regions is less than that of in the gap regions. Consequently, the asynchronous variation of the crosslinking degree between the overlap and gap regions enhanced by calcium ions will lead to an increase of the height difference between the two regions. From Fig. 4B, it can be observed that the height difference does increase with the increase of χ_Ca_ (see Fig. 4B).

For χ_Ca_ > 5 mM, the D-band periodicity and the elastic modulus of the collagen fibrils are no longer dependent on χ_Ca_; the increasing trend of the height difference affected by χ_Ca_ is not affected. These results indicate that the arrangement of the collagen molecules in the collagen fibrils are not affected by the χ_Ca_, when χ_Ca_ > 5 mM. Therefore, the increase of the height difference is attributed to the different affinities of calcium ions in the gap and overlap regions: calcium ions prefer to deposit on the overlap regions, when χ_Ca_ > 5 mM. However, the work of Nair and co-workers demonstrated that the mineral (hydroxyapatite) is deposited predominantly in the gap regions of the type I collagen fibrils^29^. The discrepancy may be due to the fact that the different factors were considered in the two studies: calcium ion and hydroxyapatite. Calcium ion and hydroxyapatite should have different mechanisms to attach with the collagen. It was also found that, for example, the ionic clusters (Ca^2+^ and [(PO_4_)_3_(OH)]^−10^) of hydroxyapatite are not arbitrarily attached to the collagen fibrils, but display correlation, that is, intergrowth of both components of the developing composite material^26^.

The variations of D-band periodicity and height difference under different χ_Ca_ indicate that the arrangement of collagen molecules in the type II collagen fibril has been altered with the emergence of calcium ions. Therefore, the variation of the mechanics of individual collagen fibril under different χ_Ca_ can be attributed to the rearrangement of collagen molecules affected by calcium ions.

## Discussion

Using AFM-based nanoindentation, we have first investigated the effect of calcium ions on the mechanics and structure of individual type II collagen fibril in this study. It was found that both of structural and mechanical characteristics of the type II collagen fibril are dependent on the concentration of calcium ions. The D-band periodicity of the collagen fibril shrinks from 68.4 ± 2.3 nm to 63.5 ± 1.0 nm, when χ_Ca_ increases from 2 mM to 5 mM. For χ_Ca_ > 5 mM, the D-band periodicity is no longer dependent on χ_Ca_. However, the height difference between the overlap and gap regions increases from 2.2 ± 0.5 nm to 3.8 ± 0.6 nm, when χ_Ca_ increases from 2 mM to 20 mM. The mechanics of the individual collagen fibril show the same trend as the D-band periodicity. In the range of 2 mM < χ_Ca_ < 5 mM, the elastic modulus of collagen fibril increases from 0.98 ± 0.02 GPa to 1.51 ± 0.03 GPa. When χ_Ca_ > 5 mM, the elastic modulus of collagen fibril is also no longer dependent on χ_Ca_. According to the chemical structure of type II collagen fibril, the chelation between collagen molecules and calcium ions is attributed to the variation of the mechanical and structural characteristics of collagen fibril.

It has been reported that the elastic modulus of individual collagen fibril (extracted from AC) and AC tissue exhibit an increasing trend with the advancement of osteoarthritis, and the length of D-band for individual type II collagen fibril decreased accordingly^16^,^22^,^30^. These mechanical and structural variations of AC and collagen fibril are together with the increase of calcium content in AC with osteoarthritis progression^30^,^31^. Based on the present study, it is suspected that calcium ions should play an important role in the pathogenesis of osteoarthritis at tissue level: the stiffening of type II collagen fibril affected by calcium ions should lead to the loss of fibril’s ductility, and so reduce fibril toughness; the type II collagen fibril therefore should have higher risk to be degraded, which is the origin of the osteoarthritis. Moreover, the present study provides solid evidences that both of the structural and mechanical characteristics of the type II collagen fibril can be tuned by calcium ions. Given that the relationship between the degradation of type II collagen fibril and the pathogenesis of osteoarthritis, our results also provide fundamental knowledge for osteoarthritis.

The detailed molecular mechanism underlying the variation of the mechanics of individual type II collagen fibril in AC remains to be further elucidated. For example, with the help of molecular dynamics simulation, detailed mechanism for the variation of collagen fibril mechanical and structural alteration under different concentrations of calcium ions may be clarified. In addition, this study has investigated only on the individual type II collagen fibril. The effect of calcium ions on other compositions of AC, such as proteoglycans, chondrocytes, should be further studied as an entirety. Not with standing its limitation, it can be anticipated that the level of calcium ions in the AC might be as a good indicator for early stage of osteoarthritis and should be rigorously considered in osteoarthritis management.

Collagen fibril is the principal composition of chondrocyte extracellular matrix (ECM), and it is known that any slight changes of ECM stiffness would have a significant influence on the normal chondrocyte functions, e.g. proliferation or migration, etc. Therefore, how χ_Ca_ within the physiological range (2–2.75 mM) affects the elastic modulus of collagen fibril and so influences the chondrocyte, is worth to be further explored.

As a consequence of the fundamental study, AFM-based nanoindentation not only has the ability to investigate the mechanics of the materials at the nanoscale level, but it also provides an opportunity to directly discover the pathogenesis of osteoarthritis. We anticipate that this method can be applied to other studies of pathogenesis of disease, such as, cardiac arrest, respiratory arrest, cataract and asteroid hyalosis.

## Method

### Sample preparation

All experimental procedures were approved by the Institutional Ethics Review Committee of Southern University of Science and Technology and the volunteer who agreed to participate signed informed consent forms. The experiments were carried out in accordance with the approved relevant guidelines and regulations. The AC used in this study was from the disposal a patient (53 years-old, Nanfang Medical School, Guangzhou, China) after total knee arthroplasty. Only the portions of AC classified as the Outerbirdge Grade 1 were selected for collagen fibril extraction. The extraction protocol of type II collagen fibril was modified based on the previous studies^32^,^33^. The AC tissues were harvested with a round punch and were cut into pieces with about 0.5 mm in thickness. The harvested AC tissues were dispersed using a homogenizer in the physiological saline (10× of sample volume) on the ice. Afterwards, the sample was centrifuged (Avanti-J-26 XPI centrifuge, Beckman Coulter) at 4500 rpm for 20 min at 4 °C for three times. After each centrifugation step, the supernatant was collected for the next centrifugation. The low temperature used here is to prevent collagen fibril from possible thermal degradation. The final supernatant was treated with CaCl_2_ (2, 3, 4, 5, 10, and 20 mM, respectively).

### AFM Measurements

In the AFM experiments, the standard glass slides were used as the substrates. Before use, the glass slides were treated by a hot piranha solution (98% H_2_SO_4_ and 35% H_2_O_2_, 7:3, v/v) for 30 min, followed by rinsing with extensive deionized (DI) water (18.2 MΩ·cm) and drying by air flow. The ideal method to investigate the mechanics and structure of collagen fibril would be to use covalent binding to a rigid surface, as described in the related studies^34^,^35^. Unfortunately, collagen fibril cannot easily be chemically modified and excessive modification of the substrate might lead to the change of the substrate stiffness, which will introduce uncertainties in the nanoindentation measurements. In addition, collagen fibril is well known for its surface adhesive properties. Thus, the simple approach of binding the collagen fibril to the glass noncovalently by physisorption was taken in this study. Therefore, to prepare the sample for the AFM measurements, a few drops of the targeted collagen fibril solution were deposited onto the pre-cleaned glass slide for 20 min. In order to remove the loosely adsorbed and minimize the number of the collagen fibril, the glass slide was rinsed with abundant DI water. Then, the sample was dried for 30 min at RT, before use in AFM measurements.

All AFM measurements were performed on an atomic force microscopy (NanoWizard II, JPK Instruments, Germany) at RT. NSG 01 tips (NT-MDT) with nominal tip radius ~6 nm were employed in this study for both structural and mechanical measurements. In advance of the AFM measurements, the laser sensitivity (*A*) was calibrated by performing several indentation tests on a clean silica, which has a much higher stiffness than that of the tip; the spring constant (*k*) of the cantilever was also calibrated using the thermal noise method^36^.

The sample was imaged under the tapping mode, with an optimized setpoint to minimum the damage and/or possible movement of collagen fibril on the glass slide. To do so, the probe was first brought into contact with a false-engagement (the probe touches the surface and immediately retracts but remains very close), and then the amplitude setpoint was slowly reduced until the probe remade contact with the surface. This ensures that there is the minimal force applied by the tip onto the collagen fibril.

The nanoindentation measurements were carried out on the individual collagen fibril based on the obtained images, as shown in Fig. 1. It was indicated that the elastic and energy dissipation properties were different for the overlap and gap regions in the collagen fibril^8^,^9^. Therefore, the nanoindentation measurements were only carried out on the overlap regions to avoid position effects on the results. Briefly, the AFM tip was driven to approach and then indented to the surface with a velocity of 0.5 μm/s until the pre-designed setpoint was reached, following by 10 s holding, finally retracted at the velocity of 0.5 μm/s. The holding segment here is to observe the time-dependent deformation, i.e., the viscoelastic deformation and/or thermal drift, during the entire indentation procedure. The height and cantilever deflection signals were simultaneously collected during the entire indentation for elastic modulus measurement. In previous studies, it was reported that the elastic modulus of collagen fibril showed a remarkable dependence on the content of water^17^. Therefore, AFM measurements were carried out at a fixed humidity of 20% under RT.

### Data analysis

**Figure 5:**
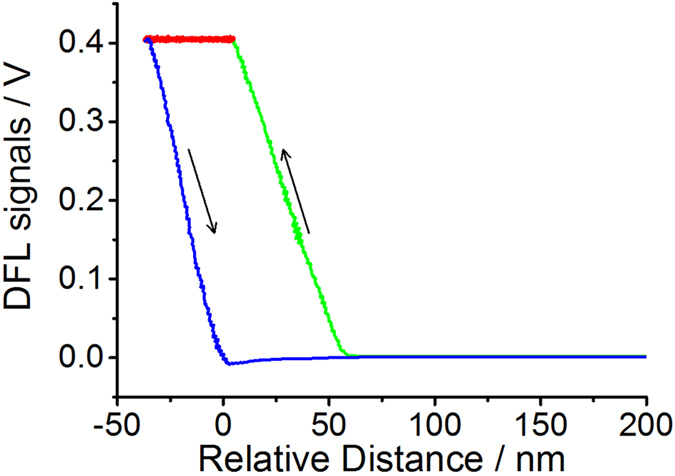
The representative curves for the displacement and cantilever deflection (DFL) signals obtained during AFM nanoindentation test performed on collagen fibril (*T* = 25 °C, RH = 20 ± 2%). The curves colored by green, red, and blue represent the approach, holding segment and retract during the AFM nanoindentation, respectively.

Figure 5 shows the typical curves for the relative displacement and cantilever deflection (DFL) signals obtained during AFM nanoindentation on the individual collagen fibril. Although the nanoindentation measurements were carried out under the dehydrated state, the significant increase of the relative distance in the holding period (see Fig. 5) revealed the significant viscoelastic deformation of collagen fibril. Therefore, the jump-rate method proposed by Ngan *et al*.^37^,^38^ was employed for the elastic modulus calculation to eliminate the viscoelastic effects. In this method, the reduced modulus (*E*_*r*_) of the tip-fibril contact can be obtained from


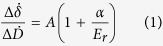


where 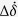 is the displacement rate jump at the onset of unholding segment, 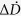 is the corresponding photodiode signal rate jump, α = *k/*2*a* is the tip-cantilever constant, where *k* is the spring constant and *a* is the contact radius. The elastic modulus of the sample can be obtained from


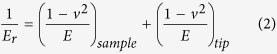


where *E* and *v* are the elastic modulus and Poisson’s ratio (*v*_*collagen*_ = 0.5^39^), respectively. *E*_*tip*_ of the tip made of single crystal silicon is typically 130–185 GPa, which is much larger than *E*_*sample*_, and so the deformation of the tip can be neglected. Therefore, the elastic modulus of individual collagen fibril can be approximated to be


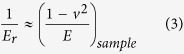


All the images of were analyzed with a commercial data processing software (JPK Data Processing Version spm-5.0.97, JPK Instruments, Germany). The height difference and the D-band periodicity of collagen fibrils were measured by using the “show an image cross section” function in the software (e.g., Fig. 1).

## Additional Information

**How to cite this article:** Pang, X. *et al*. Unraveling the role of Calcium ions in the mechanical properties of individual collagen fibrils. *Sci. Rep.* **7**, 46042; doi: 10.1038/srep46042 (2017).

**Publisher's note:** Springer Nature remains neutral with regard to jurisdictional claims in published maps and institutional affiliations.

## Supplementary Material

Supplementary Information
