# Supplementary material for: Unraveling the role of Calcium ions in the mechanical properties of individual collagen fibrils
Source: Sci Rep. 2017 Apr 5;7:46042. doi: 10.1038/srep46042 (PMC5380965; doi:10.1038/srep46042)
Supplement: Supplementary Information [file srep46042-s1.pdf]

Unraveling the role of Calcium ions in the mechanical properties of individual collagen fibrils

Xiangchao Pang[1, 3], Lijun Lin[2*], Bin Tang[1, 4*]

[1]Department of Biomedical Engineering, South University of Science and Technology of China, Shenzhen 518055, P. R. China

[2]Department of Orthopedics, Zhujiang Hospital, Southern Medical University, Guangzhou 510515, P. R. China

[3]Department of Physics and Key Laboratory of Artificial Micro- and Nano-structures of Ministry of Education, School of Physics and Technology, Wuhan University, Wuhan 430072, P. R. China

[4]ShenZhen Key Lab of Nanoimprint, Shenzhen, P. R. China

*Email: tangb@sustc.edu.cn; linlijun1@126.com

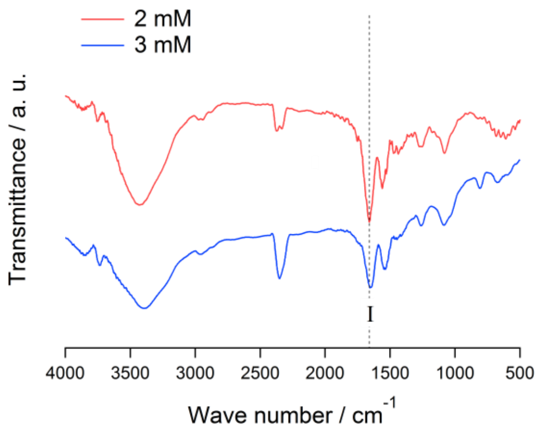

Supplemental Figure 1 FTIR spectrometry transmission spectra for the collagen fibrils under different concentrations of calcium ions.

It can be found (Supplemental Fig. 1) that there are two distinct changes on the amide I peak when the spectra of collagen/Ca^{2+}(2mM) and collagen/Ca^{2+}(3mM) are compared. First, the intensity of the amide I peak of collagen/Ca^{2+}(2mM) is higher than that of the collagen/Ca^{2+}(3mM). Second, the amide I peak, observed at 1643 cm^{-1} for collagen/Ca^{2+}(2mM), shifted to 1639 cm^{-1} for collagen/Ca^{2+}(3mM).

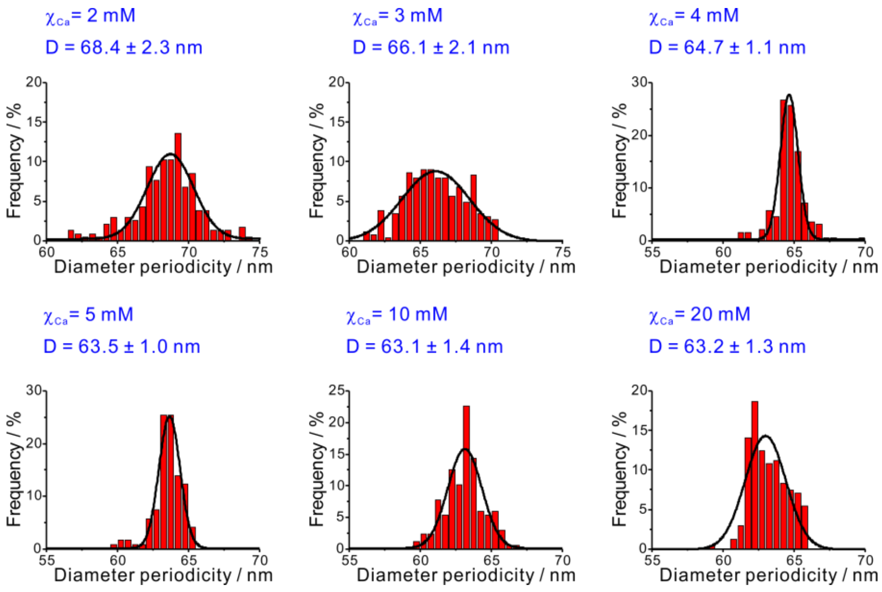

Supplemental Figure 2. The frequency histograms with the same bin of 0.5 nm of D-band period periodicity for the type II collagen fibril at different concentrations of calcium ions (χ_{Ca}). The histograms were also fitted by the Gaussian curves (black

curve). The position of the maximum of the Gaussian curve was defined as the sufficient statistics for D-band period periodicity of the collagen fibrils.

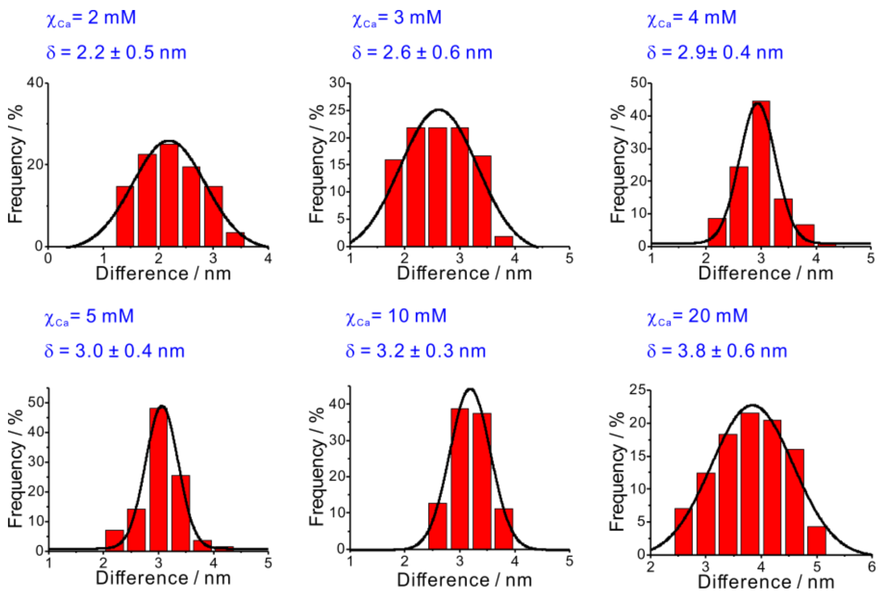

Supplemental Figure 3. The frequency histograms with the same bin of 0.4 nm of the difference between the gap and overlap regions for the type II collagen fibril at different concentrations of calcium ions. The histogram was fitted by the Gaussian curve (black curve). The position of the maximum of the Gaussian curve was defined as the sufficient statistics for the difference between the gap and overlap regions of the collagen fibrils.
